# Supplementary material for: Qualitative and Quantitative Evaluation of Rosavin, Salidroside, and p-Tyrosol in Artic Root Products via TLC-Screening, HPLC-DAD, and NMR Spectroscopy
Source: Molecules. 2022 Nov 28;27(23):8299. doi: 10.3390/molecules27238299 (PMC9739797; doi:10.3390/molecules27238299)
Supplement: Supplementary file 1 [file molecules-27-08299-s001.zip › molecules-2030972-supplementary.pdf]

Supplementary material

Qualitative and quantitative evaluation of rosavin, salidroside, and p-tyrosol in artic root products via TLC-screening, HPLC-DAD, and NMR spectroscopy

Hanna Nikolaichuk [1,2*], Marek Studziński [3] , Marek Stankevič [4] and Irena M. Choma [1,*]

[1] Department of Chromatography, Faculty of Chemistry, Maria Curie-Sklodowska University sq.3, 20031 Lublin, Poland; hanna.nikolaichuk@mail.umcs.pl (H.N.); irena.choma@mail.umcs.pl (I.M.C.)

[2] Department of Bioanalytics, Faculty of Biomedicine, Medical University of Lublin, Jaczewskiego st. 8b, 20090, Lublin, Poland; hanna.nikolaichuk@umlub.pl (H.N.)

[3] Department of Physical Chemistry, Faculty of Chemistry, Maria Curie-Sklodowska University, Maria Curie-Sklodowska sq.3, 20031 Lublin, Poland; marek.studzinski@mail.umcs.pl (M.St.)

[4] Department of Organic Chemistry, Institute of Chemical Sciences, Faculty of Chemistry, Maria Curie-Sklodowska University, Gliniana st. 33, 20613 Lublin, Poland; marek.stankevic@mail.umcs.pl (M.S.)

* Correspondence: irena.choma@mail.umcs.pl (I.M.C); hanna.nikolaichuk@mail.umcs.pl (H.N.);

Table S1. The sample list of arctic root

ID	Sample form
S1	Dry root and rhizome
S2	Dry root
S3	Supplement
S4	Rhizome
S5	United States Pharmacopean reference standard of root and rhizome (Sigma Aldrich)
S6	Dry root
S7	Supplement

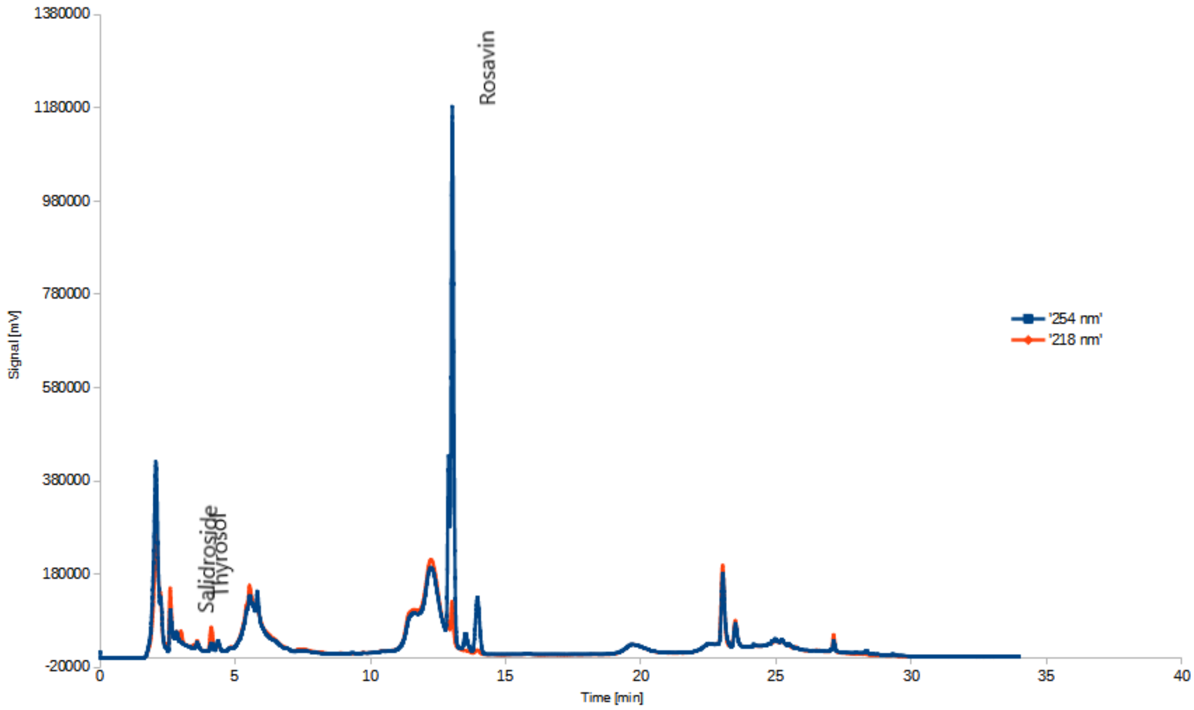

Figure S1. HPLC-DAD chromatogram of arctic root sample

(S5); at λ=218 nm (red) and 254 nm (blue).
